# Supplementary material for: Antisense Oligonucleotide-Based Downregulation of the G56R Pathogenic Variant Causing NR2E3-Associated Autosomal Dominant Retinitis Pigmentosa
Source: Genes (Basel). 2019 May 10;10(5):363. doi: 10.3390/genes10050363 (PMC6562693; doi:10.3390/genes10050363)
Supplement: Supplementary file 1 [file genes-10-00363-s001.pdf]

Supplementary data

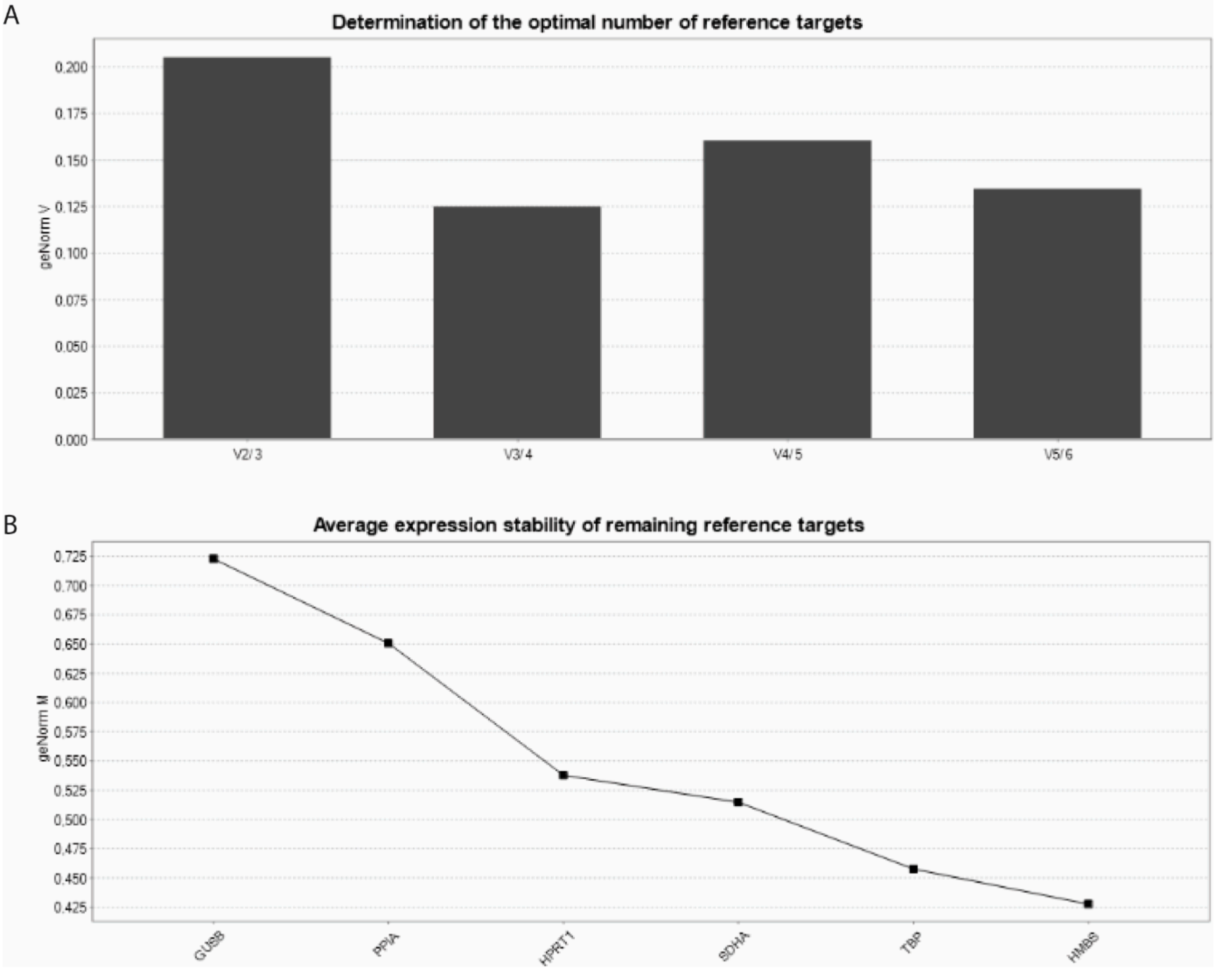

Supplementary Figure S1: GeNorm analysis to determine the optimal number and most stable reference genes. A. GeNorm V analysis showing that the optimal number of reference genes to use is three (V < 0.150). B. Genorm M analysis determining the stability of the six tested reference genes. *HMBS*, *TBP*, and *SDHA* are the most stably expressed in RPE-1 cells (lowest M values).

Supplementary Table S1: PCR primers.

	F-primer (5'-3')	R-primer (5'-3')
***NR2E3* mutagenesis primers**		
***NR2E3*_c.166G>A**	CAGCAGCAGCaGGAAGCACTATGGC	TCTCCGCACACGCGGCAC
***NR2E3* vector sequencing primers**		
**ORF_*NR2E3*_1**	GGACTTTCCAAAATGTCG	/
**ORF_*NR2E3*_2**	CAGGTCCACCTGGACAGCAT	/
**ORF_*NR2E3*_3**	GAGTGAACTCTTTCTCCT	/
**ORF_*NR2E3*_4**	/	GGGTGGTCGGAACAGGATTA
**qPCR GeNorm analysis**		
***HMBS*_expr**	GCCCTGGAGAAGAATGAA	GGTGAAAGACAACAGCATC
***SDHA*_expr**	TGGGAACAAGAGGGCATCTG	CCACCACTGCATCAAATTCATG
***HPRT1*_expr**	GACCAGTCAACAGGGGACAT	CCTGACCAAGGAAAGCAAAG
***PPIA*_expr**	AGACAAGGTCCCAAAGAC	ACCACCCTGACACATAAA
***GUSB*_expr**	AGCCAGTTCCTCATCAATGG	GGTAGTGGCTGGTACGGAAA
***TBP*_expr**	CACGAACCACGGCACTGATT	TTTTCTTGCTGCCAGTCTGGAC
***NR2E3*_expr**	GTGGACCCCACGGAGTT	CTCAGCATCACTTGGGACTG
